# The missing link-participants’ perspectives on transfer from psychosocial interventional contexts to everyday community life: a qualitative synthesis of interventional studies

**DOI:** 10.1186/s40359-021-00567-w

**Published:** 2021-04-27

**Authors:** Siv Therese Bogevik Bjørkedal, Lene Falgaard Eplov, Tom Møller

**Affiliations:** 1 CORE: Copenhagen Research Center for Mental Health. Team for Inclusion and Recovery, Gentofte Hospitalsvej 15, opg. 3A, 2900 Hellerup, Denmark; 2 CKO University Hospital of Copenhagen Rigshospitalet Dep. 8513, 2100 Copenhagen East, Denmark

**Keywords:** Rehabilitation, Recovery, Peer-support, Qualitative study, Learning, Self-management, Inclusion, Social psychiatry, Mental health

## Abstract

**Background:**

The aim of this study was to illuminate participants’ experiences with transfer in (1) Illness Management and Recovery (IMR); and (2) two programs based on peer support: Turning Points, and Learn to Tackle Anxiety and Depression (LTAD); and whether peer support within these programs influenced the process of transfer beyond the interventional context. Furthermore, we investigated participants’ experiences with a community-based intervention [Individual Placement and Support (IPS)] to explore perspectives on mechanisms that may eliminate challenges in the transfer process.

**Methods:**

The study was based on semi-structured interviews with participants with mental illness, from four different psychosocial interventions with and without peer support and across interventional settings. The material partly consists of secondary analyses of existing data sets of anonymised, transcribed interviews investigating participants' experience from two psychosocial interventions: Illness Management and Recovery (n = 15), and Individual Placement and Support (n = 12). Additionally, we conducted semi-structured interviews with persons who had participated in one of two peer-led programs: Turning Points and Learn How to Tackle Anxiety and Depression (n = 12). The analysis was guided by a hermeneutic-phenomenological approach to illuminate transfer processes and was based on the template method described by Nigel King.

**Results:**

Applying a transfer perspective on rehabilitation interventions identified everyday life situations where capacities learned during the interventions were utilized and conditions were highlighted that promoted or hindered transfer. Experiential knowledge and peer-exchange made transferal pathways between the interventional context and everyday life. Illness intrusiveness and uncertainty, together with environmental obstacles, generated transferal gaps. Individualized support could partly address these gaps.

**Conclusion:**

Findings from this qualitative study illuminate how peer-support in group-based rehabilitation interventions increased social functioning and developed better self-care strategies that can be transferred to daily life. Interventions situated in mental health settings, e.g. outpatient clinics, had limited impact on participation in broader community life. Advancing rehabilitation services in mental health may benefit from tailoring services to address illness fluctuation and combining group sessions with individualized support together with acknowledging and overcoming environmental obstacles.

## Background

Recovery, conceptualized as clinical recovery, encompassing symptom relief or remission and improved functioning, and personal recovery, referring to the process of rebuilding a satisfying and meaningful life, are the predominant goals of psychiatric rehabilitation [3, 9, 35, 36]. However, the effect of the well-known psychosocial interventions social skills training, psychoeducation, and Illness Management and Recovery (IMR) on functioning and personal recovery is unclear. Two systematic reviews from 2006 and 2008 [24, 32], found an effect of social skills training on functioning, but according to a Cochrane review from 2015 there was no significant difference between the effect of social skills training and active control groups [2]. In a Cochrane review from 2011, Xia et al. found an effect of psychoeducation on compliance and relapse prevention. A few studies included in the Cochrane review suggested a potential effect of psychoeducation on psychosocial functioning, but quality of evidence was rated as low [44]. Zou and colleagues reported similar findings in their systematic review of self-management educational programs, on relapses and prevention of re-hospitalizations. An effect on psychosocial functioning could not be confirmed, as the included studies produced conflicting results [45]. Furthermore, six RCTs testing the effect of IMR on functioning and personal recovery have yielded mixed findings [12, 14, 18, 25, 34, 39].

These interventions are based on the prerequisite that skills and knowledge acquired during the course can be transferred to everyday life beyond the interventional context, e.g. by home assignments [2, 30, 45]. However, it has been questioned whether skills learned in clinic-situated interventions can be generalized to the community [5]. Robert Lieberman described two decades ago that patients attending social skills training had difficulties generalizing skills acquired during the intervention to daily life and utilizing them outside the group [5, 19, 26]. The potential gap between clinic-based learning and the community is not unique to the psychiatric rehabilitation field. The concept of transfer stems from learning theory and refers to the process of transferring what is learned in one context to another context—to translate and apply knowledge and skills acquired during classroom-based training in real life situations [1, 17, 31].

Peer-support services (peer-support groups, peer workers) have become increasingly acknowledged in mental health sectors. In a qualitative study, Gillard and colleagues suggest that identification with a role model and building trusting relationships based on shared experiences are central mechanisms in peer-worker interventions, and these mechanisms may increase empowerment, self-efficacy and social functioning [16]. This could indicate that peer-support can potentially enhance the transfer process from interventions situated in mental health settings to everyday life in the community. Some qualitative studies of peer-workers, employed in mental health services have supported this assumption [10, 16, 37]. Storm et al. found that peer-support workers in community mental health centres often have the capacities to navigate mental health, social and primary sectors and have connections with networks in the community that can be helpful to the individual [37]. Similar findings was reported in a qualitive evaluation of a peer-led navigator program to individuals with severe mental illness [10]. Gillard et al. found in their study that peer-workers supported services users in attending activities outside mental health services, and thereby facilitated engagement in community [16].

In contrast to interventions situated in settings such as outpatient clinics and community mental health centres, is the vocational rehabilitation intervention Individual Placement and Support (IPS). The foundation of IPS is that vocational skills should not be trained in protected facilities. Instead, the person is supported directly in the workplace [7, 8]. IPS can be viewed as a response to the transfer gap as the intervention is not situated in a separate mental health environment, but in everyday life situations in the community.

The objective of this study was to explore participants’ experiences with transfer in (1) Illness Management and Recovery (IMR); and (2) two programs based on peer support: Turning Points, and Learn to Tackle Anxiety and Depression (LTAD); and whether peer support within these programs influenced the process of transfer beyond the interventional context. Moreover, we investigated participants’ experiences with an intervention situated in the community, [Individual Placement and Support (IPS)] in order to illuminate perspectives on mechanisms that may eliminate challenges in the transfer process.

## Methods

The study was designed as a qualitative, explorative and comparative study consisting of semi-structured interviews derived from four different psychosocial interventions and varying settings. The interventions were IMR, two peer-led programs (Turning Points and LTAD) and IPS. Semi-structured interviews were applied as a data-collection method because it is an available and useful method to gain insights into people’s lifeworld and learn about their perspectives regarding experiences in relation to the research topic [11, 28]. Semi-structured interviews have been applicable in a variety of contexts and research questions [27, 29, 43]. The semi structured outline in the interview guide, makes it unique among qualitative methods for the applicability it provides to explore the topic while remaining responsive to the participant [28].

The analysis was guided by a hermeneutic phenomenological approach and with a transfer lens embedded within the template method described by King [23].

### Conceptual framework

Transfer stems from adult education where the purpose is to enhance job performance [17, 31]. Transfer goes beyond *acquisition* of knowledge and skills to involve *application* of knowledge and skills. For transfer to have occurred, learned behaviour must be generalized and maintained over a period [1].

Transfer theories highlight learning as a social process, suggesting that peers can play a crucial role in transfer. Peer exchange facilitates reflections and strengthens connectedness and awareness about one’s knowledge. Input from peers can promote new opportunities to act. Peers contribute to a positive transfer climate, e.g. by impacting motivation for utilizing new knowledge and skills [1, 17].

Applying a transfer perspective on rehabilitation interventions may help to identify contexts and situations in daily life where acquired knowledge and skills are generalized, and pinpoint conditions that promote or hinder transfer. Nevertheless, fundamental differences between educational transfer and transfer in psychosocial interventions must be clarified. First, skills and strategies obtained during rehabilitation are personalized and suit the individuals’ unique situations, rather than being a pre-defined curriculum. Second, the context is not an educational situation but a broader context of daily life.

### Materials

The material partly consists of secondary analyses of existing data sets of anonymized, transcribed interviews investigating participants’ experiences from two distinct interventions: IMR and IPS [15, 21]. Moreover, we conducted interviews with persons who participated in one of two peer-led programs: LTAD or Turning Points. The unified material for this qualitative study comprised 39 interviews.

The interventions’ aims, contexts and methods are outlined in Table 1.

**Table 1:** An overview of the interventions’ aims and methods

	Illness management and recovery (IMR)	Individual placement and support (IPS)	Peer-led program 1 *(Turning points*^a^*)*	Peer-led program 2 *(“Lean how to tackle anxiety and depression”*)
Aim	To help people with severe mental illness, acquire knowledge and skills to better manage their illness as well as setting and achieving personal recovery goals	To help people with severe mental illness obtain competitive employment	Overarching aim is to assist people with mental illness in developing skills and strategies to pursue recovery	To help people with anxiety and depression acquire tools to gain control over their symptoms and to handle the challenges that accompany the illness. Additionally, to provide an opportunity where they can share experiences and thoughts with others in the same situation
Principles and/or methods	Curriculum-based psychoeducation, cognitive behavioural approaches to medication adherence, relapse prevention, social skills training and coping skills training	Focus on competitive employment, rapid job search, in-vivo support, benefit counselling, attention to client’s preference, integration with mental health teams	Peer-exchange, self-directed learning. Equal and mutual support	Curriculum-based psychoeducation, coping skills training, role modelling, peer-exchange
Duration	Nine months	Unlimited	Approximately three months depending on the course	7 weeks
Peer-support	Yes—group-based sessions	No	Yes—group-based sessions	Yes—group-based sessions
	Sessions are led by mental health professionals (e.g. nurses)		Sessions are led by peer-workers	Sessions are led by peer-workers

^a^Turning Points contains a range of courses (designed to meet different needs among the participants)

Interviews took place in surroundings according to the participants’ preferences, e.g. their home or community mental health centre. Interviews were digitally recorded and transcribed verbatim. They lasted between 45 min and 2 ½ hours. Examples of questions from the interview guides are provided in the supplemental material.

### Recruitment

Participants were recruited between 2015 and 2017. Most participants resided in Copenhagen, Denmark. Participants from the peer-led programs and from IMR received an oral or written invitation from the program’s coordinators or the researcher who attended a group meeting. The last round focused on recruiting male participants from peer-support groups because they were under-represented after initial sampling. Participants from IPS were recruited to the study by self-referral at the IPS office [15]. Accordingly, the sampling strategy for this study on transfer was predominantly based on strategic sampling.

### Method of analyses

Interviews were analysed based on King’s template analysis strategy, which combines a horizontal analysis level with an inductive approach of individual variation [23]. This strategy was chosen because the research question necessitated applying an analysis strategy that explored the individual variation and compared the perspective of groups within a specific context and with a large set of qualitative data. The initial template focused on reducing the size of the material in order to condensate on the processes of potential transfer derived from the interventions [23].

Interviews were analysed separately for each intervention and then compared across the different interventions to identify the participants’ perspectives on IMR versus peer-led programs. In Step 1, an initial template was produced with pre-defined topics: experiences linked to participation in the intervention, transfer to everyday life and experiences linked to recovery. Meaningful units were identified in the material, which was then sorted according to the defined topics. In Step 2, the meaningful units were condensed into categories and then gathered into sub-topics through an inductive approach (see example of coding in Table 2). In Step 3, the template was modified by gathering the sub-topics from the IMR study and from the peer-led programs. The volume of material was reduced. The sub-topics were read through again and, in an interactive process with the accompanying categories and meaningful units, were nuanced and re-organised into two central themes with four accompanying subthemes. In Step 4, interviews from IPS were analysed to find whether IPS could eliminate challenges in the application context identified in IMR and peer-led programs. First, an initial template with two themes, experiences of the IPS intervention and experiences of recovery, was used to identify meaningful units reflecting areas in the participants’ lives that had changed during IPS. Second, the meaningful units identified in the interviews from IPS participants were mapped into three categories, each identifying mechanisms in the IPS intervention that supported change and utilization of skills in the participants’ everyday lives. Third, in an iterative process with the transcribed interviews, and in comparison with the themes and subthemes derived from the interviews with participants from IMR and the peer-led programs, the three core categories were re-read and nuanced to a main theme reflecting how recovery is supported when the transfer challenges are avoided.

**Table 2:** Coding process (Steps 1–2)

A prior defined theme	Subthemes	Categories (to Changes in social relationships)	Meaningful units (Dealing with social anxiety while being in the group)
Transfer to daily life	Changes in social relationships	Less anxiety in social situations	“Before that, I said no to every social get-together, or I came and went within a very short time. And that changed at least. The social anxiety actually became less because it was dealt with, by being in the group"
	Changes in perceptions of life	Re-engage in contact with family	.“Every time I went home I was totally… (breathing deeply…) oh no, I can't go anymore, I've done something wrong, said something wrong, I can't… It was just like exercising—I can go next time. And I can stand it, I can handle it and the others are not a notch worse or better. And it was … it took a lot of training.”
	Factors that enhance transfer	More confident in social settings	“So I'm not sitting there all the time and thinking, am I saying something wrong? Am I doing something wrong? Am I talking too much? … Those sorts of things are indirectly trained by being in these groups”
	Factors that impede transfer	Keeping in touch with peers from the IMR sessions	
	Mental vulnerability	Meeting new friends	
	A desert islands	Dare to open (talking about symptoms, future goals)	
	Interruptions of daily rhythms and routines	More outgoing	
	Managing day-to-day life with mental illness	More present and engaged when with others	

## Results

### Participants

The empirical basis consists of 39 interviews with participants from IMR, (n = 15), two peer-led programmes—LTAD and Turning Points (n = 12)—and from IPS (n = 12). Participant characteristics are presented in Table 3.

**Table 3:** Demographic data

	Illness management and recovery	Individual placement and support	Peer-led programs^a^
Sex	6 men, 9 women	9 men, 3 women	4 men, 8 women
Age (range)	30–80	26–59	29–65
Diagnosis	Schizophrenia, Bipolar disorder	Schizophrenia, Bipolar disorder	Schizophrenia, Bipolar disorder, Recurrent depression, ADHD, Anxiety disorder
Supported living facilities	8	0	0
Work/internship/education	1	9	7

^a^Demographic data on participants from Turning Points and Learn how to Tackle Anxiety and Depression are merged and displayed in same columns

### Findings

Two central and interacting themes were identified as illuminating the transfer processes from IMR and the peer-led programs to the participants’ everyday lives in the community. The first theme, *individual experiential knowledge and peer -exchange build transferal pathways* refers to processes where knowledge and skills acquired during the sessions were applied in daily-life situations. The second theme, *Illness intrusiveness and overlooking environmental obstacles generate transferal gaps*, describes barriers and challenges in the transfer process, where acquired capacities were not utilized or where personal goals remained unrealized. The subthemes, *transfer to more profound relationships* and *transfer to better self-care,* describe distinct transfer pathways. The transferal gaps are described in two subthemes, *living with a chronic condition as an unreliable companion* and *facing real-world barriers*. The third theme emerged in IPS interviews*: A different path—the enabling role of individualized support,* with three subthemes, each reflecting enabling mechanisms; *“Having access to patient and flexible support through difficult times, “Someone to count on”* and “A natural foundation for utilizing skills and building confidence”.

**Fig. 1:**
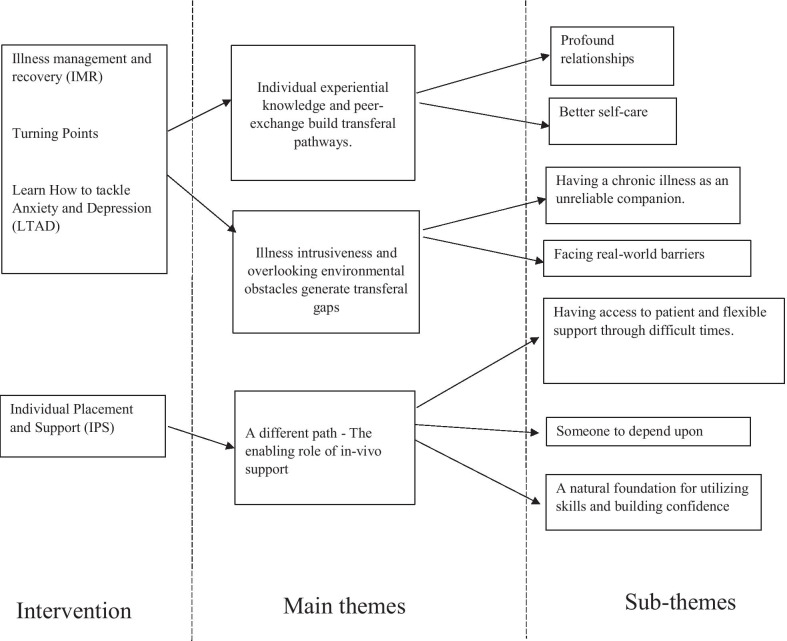
An overview of main themes and subthemes

Figure 1 illustrates central themes and accompanying subthemes

### Individual experiential knowledge and peer-exchange build transferal pathways

Participants described learning as a personal process, rather than acquisition of pre-defined knowledge and strategies. They experienced IMR and the two peer-led programs as courses where they learned about themselves and adopted new perspectives on life (Table 4, Q1 and Q2). Peer support was perceived in two ways. One, peer support was meeting other persons who struggled with mental health problems with whom one exchanged experiences and perspectives during the group sessions. For some participants, group sessions were arduous and anxiety-provoking; for others, they were a safe space (Table 4, Q7). Talking with peers about mental illness, daily matters and interests was highly valued among the participants. Several described that group conversations boosted self-confidence and gradually made them feel more present and relaxed in social situations. Two, peer support in the peer-led programs also covered peer workers, who were role models offering practical advice about how to deal with challenging daily situations (Table 4, Q6). Participants described the peer workers as someone to respect and to depend on. This required the peer workers being authentic—talking openly about their struggles with mental illness—and being a model for recovery and nurturing hope. Some participants experienced the peer workers as distanced and not sharing their own experiences; in one case, a participant perceived the peer worker as being too sick to be a role model (Table 4, Q8).

**Table 4:** Supporting quotes for themes and subthemes

	Quote nos.	
*Individual experiential knowledge and peer-exchange build transferal pathways*		
	Q1	*“In Turning Points, I learned to respect and take care of myself.”* (Female participant 1, peer-led program)
	Q2	*“I feel like I'm getting some concepts on, in my own life. But not only do I get the concepts, I also get what it means to me and how we move on from here…”* (Female participant 2, peer-led program)
Transfer to more profound relationships	Q3	*“(Before) When I had guests, I could stay for three hours, then I had to go to bed. AND that changed anyway. The social anxiety and the general anxiety were reduced because there was some processing in being in the group.”* (Male participant 2, IMR)
Transfer to better self-care	Q4	“*I probably use the action plan most, I think. As a tool*
		*When I need to do something, it is nice to have an action plan of when to do it and how much to do it and how to do it…*
		*I must prioritize my energy. Having a full-time job is plenty for me. But then I have two young children, and a wife and so it … and a family and friends and stuff like that. So, I will have to prioritize if I have an arrangement with my friends like I had yesterday for example when there was football, well I know how to do it but then I probably must be careful about plans just around that day, right? So, these are some things that one thinks about. So, I'm sure that I to some extent can live a normal life.” (laughs)* (Male participant 1, peer led program)
	Q5	*“For me the most important point was … It is OK to relax… It's like… mmm… it's like a war where you step back and it*’*s OK. Because then you can win next time.”*
		(Female participant 4, peer-led program)
The role of peer-support in facilitating transfer	Q6	*“Every time I went home I was totally… (breathing deeply…) oh no, I can't go anymore, I've done something wrong, said something wrong, I can't… It was just like exercising—I can go next time. And I can stand it, I can handle it and the others are not a notch worse or better. And it was … it took a lot of training.”* (Female participant 5, peer-led program)
	Q7	*“One of the peer workers. I sometimes sit and think, is it me you are describing or is it yourself? Which is fun, after all. And interesting. Also, because there is one here where I could ask her very specific questions. Where I could say, eh. now what if a lot of people invite you out in a week, then it will simply be too much. How do you handle it then?”* (Male participant2, peer-led program)
	Q8	“*The thing about it is that you want the teacher to be healthy. But also sick because you want to mirror yourself, but also to be healthy because they must show that the course helps. And if they are not healthy and they say that now is the fifth time I’m teaching here. Then you think. Hell!! Then it doesn't work! If you haven't recovered.”* (Male participant 3, peer-led program)
*Illness intrusiveness and overlooking environmental obstacles generate transferal gaps*		
	Q9	*“I have a lot of guilt about the things that I can't do, and I think that, or I know, it is like that for many of us, you feel ineffective, useless and impossible, right? And you take that very personally. And then you learn that you can say a lot of it really isn’t me who can’t get it right, it is the symptoms that are there and a lot of others also have the same. The symptoms. They all go round believing that it is them who can’t get themselves up from the chair. Where they sit. And then go over and do the dishes or something. They can't, and it feels very personal. When in fact, there are a lot of people who feel that way. That are struggling with that. So that's a symptom. And that means, I think, a great deal that you can throw some guilt away.”* (Female participant 5, peer-led program)
Living with chronic condition as an unreliable companion	Q10	*“But I have a melancholy side, which I think is probably because of the way I have lived my life, or relationships, or childhood or something else that makes me have such dark thoughts. Yes, it changes back and forth. I have fewer symptoms, but it still fills me up somehow. I think it would be nicer if I could do the things I feel like and the things I think I need. It can be buying Christmas presents or going to Christmas lunch or going to lectures or… Things like getting up and out the door. One day I do something and the next day I can't.”* (Female participant 2, IMR)
	Q11	*“The group suffered from the fact that there were persons there who were very good at playing therapists. Now you just … you just have to do this, you just have to do that… why don't you, and no … now you have to … take a phone call and all that… and you sit and feel so bad that you can't even go to the bathroom, right? It is kind of, we must improve our situation, all the time. And it's not always possible.*” (Female participant 5, peer-led program)
Facing real-life challenges	Q12	*“It can be a little bit… with people who are “healthy”. Healthy in inverted commas, OK? Then you can be a little, oh they are looking at me. I'm such a… and so on… So, I withdraw and… keep a low profile. Not standing out in the crowd… But feel that I do that anyway. Like there's a big elephant in the room yelling, she's mentally ill, just look at her, right?”* (Female participant 6, peer-led program)
	Q13	*“I'm not going to complain … I've gotten a lot better. And quality of life has improved. But not working and being on sick leave… Then, it doesn't matter that much anyway … so I really must practice patting myself on the back. Because I still have to say, well, it doesn*’*t matter … because I have no job.”* (Male participant 3, peer-led program)
*A different path—the enabling role of individualized support*		
	Q14	*“It's very nice because it is in many ways a kind of open offer, which means that I actually have the freedom to act.”* (Male participant 1, IPS)
	Q15	“I am really happy with my employment specialist because he, he can accommodate me and my oddities and he helps and supports and so I am allowed to try some things myself … because … if I can then it is f***ing fine, that I can myself but when it is hard for me then he supports me in it.” (Female participant 1)
Having access to patient and flexible support through difficult times	Q16	*“When I'm not feeling well, I tend to isolate myself a bit. Take care of myself, in a way … and it is nice, when he (the employment specialist) sends me an SMS … just checking up, how are you? Finding out where I am … right now … and should there be more on the table … should we have more contact … should we have less … uhhh … is it because it's going well or is it because it is not going well … so yeah, find out where I am right now and what is needed.”* (Female participant 1, IPS)
	Q17	*” When my husband left me… I felt like h… Well, we put it on hold, but we didn't give up … well, because, there were just a few months where I just didn't go out and look for jobs and these things, because I couldn't … uhhh … and it helped. I was very, very fond of her (the employment specialist)”* (Female participant 2 – IPS)
Someone to depend upon	Q18	*“The fact that I felt listened to and taken seriously and sometimes even received constructive criticism … also made me believe more in myself and therefore I also dared to do some more things. I think if I hadn’t had the support from the employment specialist that I’ve had … then I don’t think I would have dared to call those companies and ask … Can I send an unsolicited application and where should I send it? Because then I’d have been afraid of the rejection almost as soon as I introduced myself.”* (Female participant 3- IPS)
	Q19	*“He (the employment specialist) has given me a different experience. When I call him and say there is a problem, well he takes it seriously … he tries to look at it and see if he can solve it. He calls around and he sets things in motion and in many cases, well it turns out he can do a whole lot of things … which many mental health professionals have not mentioned at all as an opportunity…”* (Male participant 3, IPS)
A natural foundation for utilizing skills and building confidence	Q20	*“It (work) gives me some stability. I have problems with structure. It gives me some content … in my week … that I do something … it gives me …identity. … It gives me stability and I can combine it with the rest of my daily life … so I can have a normal week. So, I'm not just lying at home and lying on the bed. And that is important”* (Male participant 4 – IPS)
	Q21	*“I did go to college and I found out … I was actually really good at it … well … like getting As !!!! And I was both surprised and proud. And then I changed my education plan … because I found that there were some things … I was pretty good at them and I didn't know.”* (Female participant 1, IPS)
	Q22	*“During the periods when I worked, it meant a lot. My health has improved … I have had fewer admissions … shorter admissions … less medication consumption uhhh so in general just an increase in my standard of living … so from the purely psychiatric point of view, but also a question that I … that in my daily life I feel better and I have better finances, I can do more things and some things like that.”* (Male participant 2, IPS)

#### Transfer to more profound relationships

Most participants struggled with, for example, anxiety and self-contempt when engaging in social activities. A general feature of IMR and the peer-led programs was that group meetings became a safe arena to practice communication skills and build confidence that was transferred to other social situations in the participants’ daily lives. Talking with peers, during the course had strengthened the participants’ relationships with family and friends. They described becoming more comfortable and present in the company of their loved ones, becoming better at expressing their thoughts and becoming careful listeners (Table 4, Q3). However, participants also expressed some distance to family members who they felt could not fully understand the illness’s intrusiveness, for example, not being able to eat out, or attend family gatherings.

Acquired social skills and confidence was also transferred to encounters with mental health professionals. Some participants, mainly from the peer-led programs, described encounters with professionals as being difficult, for example, to explain one’s situation. Engaging with peers enhanced assertiveness and provided inspiration for new reflections and agendas used in meetings with therapists or councillors.

#### Transfer to better self-care

A unique feature of the peer-led programs was that participants learned self-care strategies that were transferred to everyday life. They utilized the strategies to make plans that complemented their energy levels, carefully choosing what arrangements to engage in (Table 4, Q4). One man from Turning Points highlighted the relief he felt from learning new ways to decline invitations without fear of disappointing friends and family. Others learned strategies to tackle anxiety attacks, such as choosing a seat close to an exit, strategies enabled them to attend concerts and lectures, activities that used to be impossible before joining the course. The peer workers’ own experiences were the main source of learning strategies, which were often innovative and not typically “by the book”. Participants could mirror themselves in the peers’ narratives and found the advice and tips more useful than that from health care workers, which was often perceived as unrealistic or fit only for an ideal world. Participants greatly valued the peer workers’ human touch. Two women from the peer-led programs said that the most important thing they had learned during the course was to go slowly, accept the bad days and to take care of themselves (Table 4, Q5).

### Illness intrusiveness and overlooking environmental obstacles generate transferal gaps

Participants from IMR and the peer-led programs identified conditions that undermined transferring what they had learned during the course to everyday life. They struggled with disabilities that disrupted daily living and hampered opportunities to pursue personal goals. The inability to carry out everyday activities and do what people around them took for granted caused shame, guilt and feelings of worthlessness. Some did not perceive the group sessions as a learning situation per se, rather a breathing space and a haven, where they could share experiences with peers and meet understanding and acceptance (Table 4, Q9).

Participants had various reasons for attending the courses. Often, the need to share experiences and meet peers was greater than the need to learn new skills and strategies. Some women from LTAD described the content as “nothing new under the sun”. Participants expressed a major shift from learning new strategies or voicing personal goals in the group and subsequently acting on them. The interventions had limited transfer to the broader domains of daily life, such as handling practical activities—e.g. shopping or ordering a taxi—work, education and joining community activities. The transferal gaps are described in two subthemes: *Living with chronic illness as an unreliable companion* and *Facing real-world barriers.*

#### Living with chronic illness as an unreliable companion

Most participants struggled with unpredictable, fluctuating and intrusive symptoms; anxiety; hallucinations; and lack of impetus and energy, disrupting their everyday lives. The symptoms made them cancel plans, struggle with practical tasks, caused difficulties leaving their homes or enjoying interests. In general, participating in IMR or the peer-led programs did not alter the intrusive impact of their symptoms. Living with mental illness was like living with an erratic and unreliable companion (Table 4, Q10). Many described the process of making changes as arduous or anxiety provoking. Fear of failure prevented some participants from setting goals or trying new activities. Home assignments were perceived differently among the participants. For some, these assignments supported them in pursuing personal goals during the course, such as taking up new activities or altering morning routines. Others felt the assignments were an imposition. One woman criticized LTAD for assuming it was always possible to improve one’s situation and that there should be constant progress. Overall, she agreed that one could actively work on improving the situation, but she also emphasized that this was not always possible (Table 4, Q11). Consequently, the process of transferring new capacities to the context of daily lives and putting actions behind words was often challenging and sometimes unfeasible.

#### Facing real-world barriers

Being detached from the community was a common experience among participants. They felt that they were living strange lives that were out of balance. Struggling with an invisible and disabling illness made the participants feel estranged from their loved ones and unaccepted in society (Table 4, Q12). Some had lost friends during the years of struggling with illness, leaving them isolated and lonely. A few experienced a vacuum after the course had ended. One woman compared the time after finishing LTAD to her childhood memories of the greyness and emptiness in the days after Christmas Eve. Activities, such as going to concerts, were challenging when one had to do it alone. One woman from IMR said that she felt like a desert island and that society saw her as an illness and not a human being.

Finding employment was a main goal for many participants. Attending the peer-led courses had inspired some participants to become peer workers themselves. Others expressed disappointment at not finding employment after they had completed the course (Table 4, Q13). Learning new strategies and gaining more personal confidence was not enough to obtain work as many participants lacked work experience or professional training due to years of struggling with mental illness. Receiving social benefits had considerable consequences for the participants’ daily lives, for example, not being able to repay loans or take vacations.

The interviews revealed that skills and knowledge could be transferred to some extent from the interventional context to the participants’ daily lives in the community, and that peer support facilitated this. However, the findings also identified transferal gaps. Utilizing new strategies and pursing personal goals were hampered by the intrusive and disruptive nature of the mental illness and environmental conditions such as loneliness, financial matters, and labor-market demands. In the next section, these findings are compared with the experiences of participants from IPS, where there are no transfer challenges.

### A different path—the enabling role of individualized support

Three enabling mechanisms in individualized support emerged in the interviews with IPS participants. Firstly, the continuous and flexible nature of the support offered by the employment specialist (ES) enabled participants to pursue vocational goals whilst dealing with relapses, struggling with symptoms etc (Table 4, Q14 and Q15). Secondly, not being alone while searching for jobs, and having someone who believed in them and supporting them encouraged the participants to take risks and face challenges in the job searching process. Thirdly, utilizing skills and knowledge while searching for jobs, working or studying built confidence and new capacities. Hence, engaging in real-life activities was a natural foundation for personal growth and recovery. The three mechanisms identified in the IPS interviews are captured in the subthemes; “*Having access to patient and flexible support through difficult times*” and “*Someone to count on*” and “A natural foundation for utilizing skills and building confidence”.

#### Having access to patient and flexible support through difficult times

The IPS’s approach of flexibility and unlimited time allowed participants to apply for jobs when it suited them. Participants valued the continuity of the ESs support—that they were there for them, through hospitalizations, worsening illness and life-events. They experienced the ESs being sensitive to their needs, offering the type of support appropriate for the situation (Table 4, Q16). This also involved postponing job searching, if necessary (Table 4, Q17). Participants experienced the support as highly individualized and appropriate for their specific needs, enabling them to pursue long-term goals such as completing an education or obtaining work.

#### Someone to depend on

Participants described job searching as a demanding process with both good and bad experiences. They highlighted the core ingredient in IPS: to have someone “on their side”, someone who they could relied on (Table 4, Q18). The type of support depended on the participants’ situation. It included having the opportunity to share both successes and frustrations, update CVs, and search for jobs together as well as receiving an SMS with a “good luck” greeting on the first day of a new job. The ES’s ability to navigate the complex welfare system, providing access to social services, was highly valued among the participants. So was the experience of being met as person, rather than as a patient or client. Participants valued the ES allowing them take initiatives and responsibility, and face challenges, for example, when contacting potential employers. The relationship with the ES boosted participants’ confidence and determination to take chances and try out new situations while applying for work (Table 4, Q17).

#### A natural foundation for utilizing skills and building confidence

Being a student or employee promoted a positive self-image, an identity beyond mental illness, an outlook of a better future, and a more solid economic basis from which to enjoy life (Table 4, Q20 and Q21). Some participants described a “domino effect”: engaging in education or work influenced other aspects of their daily life, such as taking up hobbies, improving daily routine or doing more daily activities such as cooking meals (Table 4, Q19). Others described strategies and tips that they had adopted from the ES that enhanced their self-care in terms of balancing activity and rest.

## Discussion

In this study, we analysed interviews with participants from IMR and two peer-led programs, to identify whether and, if so, how transfer from the interventional context to daily life occurred and how peer support influenced transfer processes. Transfer gaps and transfer paths co-existed in most participants’ narratives. They experienced group sessions as a safe place to practice social skills, build confidence and reconnect to goals and dreams. The group became a personal learning forum where agendas and aspirations—combating loneliness, finding interests and re-engaging in work, education or community activities—were nurtured and strengthened during the course. The capacities acquired during the course could to some extent be transferred to social situations beyond the learning contexts, allowing the participants to become more present, relaxed and confident around their loved ones, and more assertive in encounters with professionals. Transfer theories highlight that similarities between contexts increase the possibility of transfer [1]. This might explain why transfer related to social functioning was the most prominent theme across interviews, while transfer connected to functioning in other areas, for example, finding a job or going grocery shopping, generally was not identified. Several participants had difficulties in their daily lives, for example, when doing daily tasks, finding work, or engaging in community activities, that remained unchanged after the course had ended. All participants described how intrusive and disruptive symptoms impacted their lives, their relationships, their opportunities for living well and doing what they wanted. In our study, illness management did possibly relieve, but certainly not eliminate the disabling impact of symptoms. This finding may modify a central rationale behind self-management interventions, that better illness management leads to living more functional lives [6, 30, 45] and add perspectives to why effectiveness of psychoeducational programs on psychosocial functioning might be hard to determine in the systematic reviews by Xia et al. and Zou et al. [44, 45]. Notably, in the peer-led programs, learning new self-care strategies, e.g. prioritizing energy or practicing self-compassion, was highly valued and could be transferred to the participants’ daily lives. Interestingly, strategies and skills obtained during the course were not from the curriculum but from the peer-workers’ personal experiences. Transfer theories underline that transfer can be enhanced by the teacher’s insight and experiences regarding the student’s context. Accordingly, examples and practical and concrete explanations facilitate the transfer process [1]. That could explain why many participants found the strategies and skills learned from peers more useful and applicable than the advice and input from mental health professionals. This has been addressed in a review by Davidson and colleagues, suggesting that peers introduce new ways of using experiential knowledge in negotiating day-to-day life, not only with the illness but also with its social consequences such as poverty and stigma [13].

Living with disability and vulnerability was perceived as a lonely experience for the interviewees, who felt disabled and estranged. Many described feeling more comfortable among their peers than among so-called healthy people and experienced the group as a place for connectedness difficult to experience elsewhere. This corresponds with findings by Van Langen et al., where participants in IMR groups described feeling deviant and alienated in their own social environment. They described the group sessions as a refuge from daily life, where they felt safe, accepted and understood [42].

The “Missing Link” in our study refers to the gaps in the transfer processes and may be related to putting too great an emphasis on the individual process of recovery during the interventions. Our study shows that having hopes, aspirations and confidence may not be sufficient to ensure recovery if the person’s environment does not provide opportunities for continuous support, inclusion and community participation. It has been suggested that social aspects of recovery have been neglected [22, 33, 41]. Recovery is both an individual and a social process. Borg and Karlson describe important aspects of recovery as being supported and valued for the person one is, engaging in meaningful activities and being welcomed and accepted by other people and in various social settings, overcoming stigma and no longer being discriminated against due to one’s health condition [22]. Recovery unfolds in environments and situations that provide opportunities for nurture, challenges and recognition. Hence, recovery is not only an individual matter, it highly depends on the person’s network, access to support and opportunities in the community. In contrast, the participants in our study often felt alienated, deviant and isolated in the community. Correspondingly, in a study including 27 countries, Thornicroft found that among participants negative discrimination was experienced by 47% when making or keeping friends, by 29% when finding a job, and by 29% in keeping a job [40].

In IPS interviews, we found three enabling mechanisms of individualized support: the unlimited time and flexible support, having someone to depend upon, and real-world activities as arenas for personal growth and recovery. To some extent in-vivo support addresses some of the gaps embedded in the missing link. First, the support was tailored to the participants taking their life situation into account; the intensity, duration, and type of support was planned and delivered in close collaboration with the participants. Many participants stressed the importance of being met as whole persons with vulnerabilities and strengths. Correspondingly, in a qualitive study with IPS participants in Sweden, Areberg et.al. found that having someone on one’s side—someone who validates ones wishes and needs—and having opportunities for utilizing one’s own skills and competences brought hope and meaning to the process of searching for work [4].

### Implication for clinical practice

Group sessions can build dreams, goals, aspirations and self-growth and thereby a motivational source in the recovery process. Our findings underline the necessity of providing such forums in recovery-oriented services; they also highlight the group sessions’ shortcomings because the skills and confidence gained during the course to support recovery in the broader aspect of daily life and community participation are difficult to apply in other contexts. Clinicians and policy makers should be aware of this transferal gap. Our findings show how manifestation of mental illness can shape a daily life dominated by long-term disability and fluctuating invasive problems, and that psychiatric disabilities are often experienced as shameful and deviant. This raises the question of whether an underlying assumption of constant progress in rehabilitation services may put an unintentional pressure and risk of self-blame on the service user. Our findings identify a need to broaden approaches and pay more attention to the social sides of recovery when developing and advancing psychosocial rehabilitation services. First, there seem to be possible benefits of combining group-based interventions with individualized support. Second, more attention should be paid to the person’s own environment, for example, by creating opportunities for participation and enhancing places and communities that foster inclusion and connectedness.

### Strengths and limitation

This study applies secondary analysis of data from two previous qualitative studies, investigating participants’ experience of recovery during IMR and IPS, respectively [15, 21]. The research questions and methodology were compatible with our study, which made it partially possible to analyse the material in a transfer perspective [20, 38]. The interview method provided insights into the participants’ experiences with the interventions and enabled new understandings of whether and how there was a transfer from the interventional setting to everyday life. The researcher was sensitive about data being embedded in different contexts [20, 38]. Since secondary and original researchers were employed in the same institution, it was possible to provide additional information about the background and clarify any questions regarding the context [38]. Secondary analysis has limitations. First, it is not possible to recruit participants consistently until saturation has been reached. Hence, the sample needs to be sufficiently large. Although patterns became clear across interviews, there is a possibility that adding more interviews would have generated new informative data and more nuances of the findings. Second, member checking was not possible. The secondary researcher could not question the informants or conduct second interviews. We aimed to overcome this limitation by discussing the findings with an advisory board of mental health service users, who served as a source of validation [38].

The recruitment strategy favoured participants who were either participating in the intervention at the time of interview or who had completed the course. This question the transferability of the results as the perspectives of participants who dropped out during the interventions are not represented in this study. Applying a theoretical framework with a priori defined themes may have led to central themes in the interviews important to the participants and their recovery being overlooked. However, we attempted to make the codes as open as possible to capture the essence in interviews related to the study question.

## Conclusions

Our findings highlight the gaps and paths between obtaining new knowledge and skills during group-based rehabilitation interventions, IMR and peer-support groups and applying and utilizing these capacities in the community. Group sessions have advantages regarding enhancing motivation and strengthening social skills and strategies for self-care. Experiential knowledge and sense of connectedness in peer support increases the transfer process. However, overlooking mental illness intrusiveness and uncertainty, and the environmental obstacles generates transferal gaps between interventional context and everyday life in community. Group sessions have shortcomings when it comes to enabling participation in, community life, work and education, and in carrying out. practical activities. Our results suggest that individualized support can partly counter the challenges identified in the transferal gap, providing the support is authentic, flexible and tailored to the person’s needs and situation. Importantly, our findings show a need to address the social aspects of recovery in order to advance and increase the opportunities for participation in the community as well as in work and education if individuals struggling with mental illness are to be supported successfully in their recovery. Consequently, future interventional studies in the field of psychiatric rehabilitation should address structural barriers for social inclusion and community participation that people with mental illness face. This includes a closer scrutiny on physical and social attributes in the individual’s environment, such as places, neighbourhood and presences of other people, and on underlying subtle processes embedded in the person’s community, such as social interactions, cultural meanings and shared values. In this context, and in the view of the results in this study, more research on environmental enhancements and accesses to community resources, for supporting recovery and promoting health and wellbeing is warranted.

## Data Availability

The datasets analysed in this study are not publicly available, due to Danish law that prohibit data sharing.

## Abbreviations

IMR: Illness management and recovery
IPS: Individual placement and support
LTAD: Learn how to tackle anxiety and depression
